# Immunomodulation in Heart Failure with Preserved Ejection Fraction: Current State and Future Perspectives

**DOI:** 10.1007/s12265-020-10026-3

**Published:** 2020-05-22

**Authors:** Elise L. Kessler, Martinus I.F.J. Oerlemans, Patricia van den Hoogen, Carmen Yap, Joost P.G. Sluijter, Saskia C.A. de Jager

**Affiliations:** 1 Laboratory of Experimental Cardiology, Cardiology, UMC Utrecht Regenerative Medicine Center, University Medical Center Utrecht, Utrecht, Netherlands; 2 Netherlands Heart Institute, 3511 EP Utrecht, Netherlands; 3 Circulatory Health Laboratory, Utrecht University, University Medical Center Utrecht, Utrecht, Netherlands; 4 Department of Cardiology, University Medical Center Utrecht, Utrecht, Netherlands; 5 Center for Translational Immunology, University Medical Center Utrecht, Utrecht, Netherlands

**Keywords:** HFpEF, LVDD, Immunomodulation, Inflammation, Clinical trials, Preclinical models

## Abstract

The heart failure (HF) epidemic is growing and approximately half of the HF patients have heart failure with preserved ejection fraction (HFpEF). HFpEF is a heterogeneous syndrome, characterized by a preserved left ventricular ejection fraction (LVEF ≥ 50%) with diastolic dysfunction, and is associated with high morbidity and mortality. Underlying comorbidities of HFpEF, i.e., hypertension, type 2 diabetes mellitus, obesity, and renal failure, lead to a systemic pro-inflammatory state, thereby affecting normal cardiac function. Increased inflammatory biomarkers predict incident HFpEF and are higher in patients with HFpEF as compared with heart failure with reduced ejection fraction (HFrEF). Randomized trials in HFpEF patients using traditional HF medication failed to demonstrate a clear benefit on hard endpoints (mortality and/or HF hospitalization). Therefore, therapies targeting underlying comorbidities and systemic inflammation in early HFpEF may provide better opportunities. Here, we provide an overview of the current state and future perspectives of immunomodulatory therapies for HFpEF.

**Electronic supplementary material:**

The online version of this article (10.1007/s12265-020-10026-3) contains supplementary material, which is available to authorized users.

## Introduction

Heart failure (HF) with preserved ejection fraction (HFpEF) accounts for ≈ 50% of all heart failure (HF) patients (≈ 6.2 million American adults with HF from 2013 to 2016) with increased prevalence in the elderly population (> 70 years) and women (> 80% of all HF diagnoses) [1–4]. HFpEF is diagnosed when HF symptoms are present in combination with signs of left ventricular diastolic dysfunction (LVDD) and elevated levels of natriuretic peptides are evident, but left ventricular ejection fraction is preserved (LVEF ≥ 50%) [5]. HFpEF is accompanied by a plethora of comorbidities, including hypertension, obesity, type 2 diabetes mellitus (T2DM), and coronary artery disease, which contribute to increased morbidity and mortality [3, 6–8]. Some comorbidities cause a chronic pro-inflammatory state, leading to structural and functional alterations of the vasculature and myocardium ultimately resulting in the clinical syndrome of HFpEF [6, 9–12]. Persisting “chronic” inflammation is one of the hallmarks of disease progression, with disturbances in humoral anti-cardiac autoimmunity as one of the involved pathways [8, 11, 13]. HFpEF is associated with elevated levels of cytokines, chemokines, and endothelial adhesion molecules promoting infiltration of activated inflammatory cells into the heart [8, 9]. Altogether, these data suggest that therapies targeting inflammation could be more effective in HFpEF than current treatment options. This review summarizes traditional therapies for HFpEF and its comorbidities with a chronic inflammatory profile and discusses the current state and future perspectives of immunomodulation, based on experimental and preclinical models.

## Current Treatment Options for HFpEF

### Guideline-Based HFrEF Treatment

Thus far, randomized trials in HFpEF patients using traditional HF medication failed to demonstrate a benefit on mortality and hospitalization for HF (HHF) [11]. Despite proven efficacy in HFrEF, β-blockers (nebivolol), angiotensin receptor blockers (ARB; candesartan, irbesartan), ACE inhibitors (ACEi; perindopril), and digoxin showed a neutral effect on mortality and hospitalization in the HFpEF population. Supplementary Table 1 provides an overview of important phase III clinical trials for HFpEF. Given the important role of inflammation in HFpEF [14], it is striking that effects on the inflammatory state are in most cases not reported. Only the TOPCAT trial measured high-sensitive C-reactive protein (hs-CRP) [15], which were not affected by treatment and may have contributed to the lack of benefit.

### Comorbidities with a Chronic Inflammatory Profile

Trials targeting systemic inflammation in HFpEF are currently lacking. Therefore, we summarize preclinical research and ongoing trials focusing on hypertension and T2DM, which have a known inflammatory phenotype.

#### Hypertension

Hypertension is the most common comorbidity (> 65%) in HFpEF and associated with inflammation [16]. Angiotensin II is responsible for hypertension-induced inflammation and subsequent myocardial and vascular damage [17]. However, commonly used drugs for hypertension (ACEi, ARB) showed a neutral effect on mortality or HHF (Supplementary Table 1). Data on calcium channel blockers in HFpEF patients are lacking, although trials in HFpEF patients with pulmonary hypertension are ongoing (identifier: NCT03153111, NCT03620526, and NCT03043651).

#### Type 2 Diabetes Mellitus

Approximately 45% of all HFpEF patients have T2DM, and T2DM patients are twice as likely to develop HF [18]. T2DM increases morbidity and mortality in HFpEF patients [19, 20]. Several mechanisms could explain this increased risk. Insulin resistance enhances free fatty acid metabolism and reduces myocardial glucose uptake, leading to increasing amounts of toxic intermediates and reactive oxygen species (ROS) [21]. Subsequent secretion of pro-inflammatory cytokines by the myocardium and epicardium attracts macrophages impairing myocardial and vascular function [22]. Furthermore, hyperglycemia increases sodium retention, and T2DM patients in general have increased neurohumoral activation [23].

##### Metformin

Metformin decreases hepatic glucose production, increases tissue insulin sensitivity, and is associated with improved cardiac outcome based on experimental models. Metformin attenuated LV remodeling in a hypertensive rat model by inhibiting aldosterone-induced fibroblast activation [24]. A study assessing the effect of metformin on functional capacity and hemodynamic parameters in HFpEF patients with pulmonary hypertension is ongoing (NCT03629340).

##### Thiazolidinediones

Thiazolidinediones (glitazones) stimulate fatty acid storage in adipocytes, thereby increasing glucose utilization and lowering serum glucose level. However, they may cause sodium and water retention leading to congestion [5]. Dipeptidylpeptidase-4 inhibitors (DPP4i; gliptins) and glucagon-like peptide-1 (GLP-1) receptor agonists, which increase insulin release and prolong its action, are suggested to worsen HF although the exact mechanism remains unclear [25, 26].

##### Sodium-Glucose Cotransporter 2 Inhibitors (SGLT2i)

This novel class of drugs showed benefits beyond glycemic control in preventing HF in T2DM patients. SGLT2i reduces renal glucose reabsorption and increases urinary glucose excretion, next to restoration of sodium delivery, thereby increasing diuresis [27–29]. The EMPA-REG OUTCOME trial was first to show a 38% reduction in cardiovascular mortality in high-risk patients with T2DM [27, 29]. The phase III clinical trial testing empafligozin in HFpEF patients with and without T2DM (EMPEROR-PRESERVED; NCT03057951) is still ongoing [30]. Furthermore, an ongoing study testing dapafligozin in HFpEF patients (PRESERVED-HF; NCT03030235) is assessing changes in N-terminal prohormone of brain natriuretic peptide (NT-proBNP) levels as primary outcome.

##### Peroxisome Proliferator-Activated Receptors (PPAR-γ) Agonists

PPAR-γ agonists (glitazones) reduce inflammation, oxidative stress, and hypertrophy in adipocytes and cardiomyocytes [31]. In a HFpEF rat model (8%NaCl-diet), pioglitazone attenuated the development of LV fibrosis and stiffening and prevented development of HFpEF [32]. Another study with obese rats showed that pioglitazone attenuated LV hypertrophy, fibrosis, and LVDD [33]. An observational study with pioglitazone demonstrated improvement in diastolic filling properties in patients with T2DM [34].

#### Obesity and Lipid Metabolism

Obesity and hyperlipidemia are important comorbidities in HFpEF and linked to systemic inflammation [35]. Weight loss improved exercise capacity and led to a reduction in hs-CRP [36] and reduced pulmonary artery pressure leading to an improved hemodynamic profile [37]. Due to the metabolic derangement in obesity, hyperlipidemia is frequently present leading to the prescription of statins, which gained interest for their beneficial pleiotropic effects including improvement of endothelial dysfunction and antioxidant- and anti-inflammatory properties in cardiovascular disease [38]. Importantly, statins also exert a direct immunomodulatory effect by reducing expression of major histocompatibility complex II (MHCII) and T cell response [39]. Although currently not recommended as standard therapy, statins might improve outcome in HFpEF [40]. In an experimental HFpEF rat model, statins suppressed concentric remodeling and decreased collagen synthesis [41]. A recent post hoc analysis of the TOPCAT trial and a large registry (> 113.000 patients) showed associations between statin use and all-cause and cardiovascular mortality [42, 43]. Taken together, statins hold great potential, but large clinical trials are necessary to show improvement in mortality and/or hospitalization.

## Immunomodulation in HFpEF

**Fig. 1:**
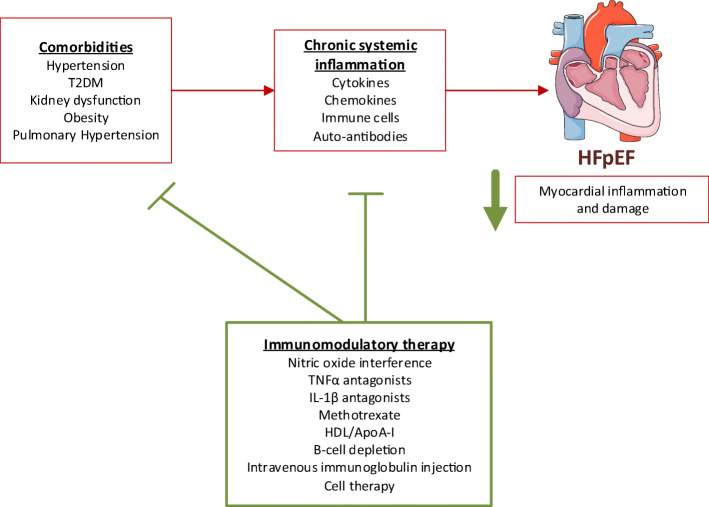
Pathophysiological mechanisms of heart failure with preserved ejection fraction (HFpEF) as targets for therapy. Comorbidities, such as hypertension, type 2 diabetes mellitus (T2DM), and obesity, lead to chronic systemic inflammation and subsequently HFpEF, associated with myocardial inflammation and damage (red). Immunomodulation targeting either comorbidities or underlying disease mechanisms (green), which is the focus of this review, can decrease myocardial inflammation and damage and are currently under evaluation

Although inflammation is a major driver in the pathophysiology of HFpEF, trials primarily focusing on systemic inflammatory pathways in HFpEF are currently lacking. Targeting specific components of the inflammatory process might decrease cardiac remodeling and benefit cardiac function. Based on experimental data, important novel therapeutic options are discussed below and summarized in Fig. 1.

### Nitric Oxide Signaling

Normally, NO is produced by cardiac microvascular endothelial cells resulting in activation of soluble guanylate cyclase (sGC) in cardiomyocytes and vascular smooth muscle cells. sGC in turn produces cyclic guanosine monophosphate (cGMP), which regulates protein kinase G (PKG) [44]. However, in HFpEF this pathway is often defective, and low PKG activity results in cardiomyocyte hypertrophy and can eventually lead to LVDD [9]. Interference with this pathway could provide benefits for vascular and myocardial remodeling, including attenuation of hypertrophy, fibrosis, impaired cardiac relaxation, and eventually LVDD [9].

#### Phosphodiesterase-5 Inhibitors (PDE5i)

PDE5i inhibit breakdown of cGMP levels, thus activating protein kinase G, which resulted in decreased LV/cardiomyocyte stiffness and LV relaxation time and prevention of HFpEF in Zucker diabetic fatty rats [44]. However, in the RELAX trial, the PDE5i sildenafil did not alter exercise capacity nor improves clinical status in HFpEF patients [45].

#### Nitrate and Nitrite

Nitrate and nitrite increase NO bioavailability, contributing to vascular integrity and function. In the NEAT-HFpEF trial, nitrate isosorbide mononitrate caused HFpEF patients to be less active and no QoL improvement was observed [46, 47]. In the INDIE-HFpEF trial, inorganic nitrate was inhaled, which again showed no improvement in exercise capacity and QoL [48], which could partially be due to the short-acting nature of nebulized nitrite [46].

#### Soluble Guanylate Cyclase Stimulators (sGCs)

The sGCs riociguat has proven anti-fibrotic, anti-proliferative, and anti-inflammatory effects [49]. However, in the DILATE-1 trial, treatment of HFpEF patients with pulmonary hypertension did not change mean pulmonary artery pressure but increased stroke volume and decreased systolic blood pressure and right ventricular end-diastolic area. In the SOCRATES-PRESERVED trial, treatment with the sGCs vericiguat in HFpEF patients showed improvements in QoL, even though natriuretic peptide levels and left atrial volume did not change [50].

### TNF-α Antagonists

Pro-inflammatory cytokines, including TNF-α, IL-1, and IL-6, can lead to progressive LV dysfunction and remodeling, pulmonary edema, and cardiomyopathy [51]. TNF-α promotes LV remodeling mostly through alterations in the extra cellular matrix (ECM) [52]. Rats treated with TNF-α (dosage reflecting levels in HF patients) presented with time-dependent changes in LV dimension accompanied by a progressive degradation of the ECM [51] and in an experimental rat model with streptozotocin-induced diabetic cardiomyopathy, a monoclonal antibody against tumor necrosis factor alpha (TNF-α) as well as an interleukin converting enzyme (ICE) inhibitor protected from myocardial inflammation and fibrosis [53, 54]. Additionally, transgenic mice overexpressing TNF-α develop progressive LV dilation and congestive HF [55].

#### PDEis and Inhibition of TNF-α Production

The PDEi pentoxifylline improved LVEF and reduction of TNF-α and Fas/Apo-1 in HFpEF. However, TNF-α was still increased compared with healthy individuals and CRP was not measured [56]. Thalidomide inhibits production of TNF-α and other inflammatory mediators and is clinically used in Crohn’s disease, despite unclear mechanism of action [57–59]. In HFrEF patients, LVEF and QoL were improved, and a significant decrease in LV end-diastolic volume and heart rate was observed [10]. This was accompanied by decreased protein expression of the matrix degrading matrix metalloproteinase-2 (MMP-2) [60]. Trials of thalidomide in HFpEF patients have yet to be conducted.

#### Aldosteric Antagonists for the TNF Receptor and (Chimeric) Antibodies

Studies in HF patients with the aldosteric antagonists for the TNF receptor etanercept showed improved QoL, increased 6-min walk distance, and improved LVEF [59]. However, two multicenter trials using different dosages of etanercept (RECOVER and RENAISSANCE) were terminated prematurely because of lack of improved clinical outcome in patients. This may be related to etanercept properties that enable stabilization of TNF-α and increase its bioactivity in vivo. Increased circulating levels of biologically active TNF-α in HF patients might therefore contribute to worsening of HF symptoms [59]. The chimeric antibody infliximab neutralizes TNF-α and is effective in Crohn’s disease and rheumatoid arthritis. Infliximab was tested in HF patients (ATTACH) and resulted in increased mortality and HHF, possibly through complement fixation in the heart [61].

### IL-1 Antagonists

IL-1β is primarily produced by inflammatory cells and a product of cytoplasmic innate immune complexes, the inflammasomes [62–64]. Crohn’s disease, psoriasis, and rheumatoid arthritis can all be treated with IL-1 antagonists [63]. In a mouse model of acute myocardial infarction (MI), IL-1β antagonists improved LV diastolic function [65]. The IL-1 receptor antagonist anakinra showed to reduce systemic inflammation and improve aerobic exercise capacity in a pilot trial (D-HART) in HFpEF patients with high inflammatory risk (CRP > 2 mg/L) [66]. However, in the subsequent phase II trial (D-HART2), anakinra failed to improve cardiac output, reflected by a lack of benefit on peak oxygen consumption and ventilatory efficiency, despite a reduction in CRP and NT-proBNP, and improvement in exercise time and QoL [40, 67].

#### Decrease of Uric Acid Production

Uric acid is known to predict mortality and the need for heart transplantation in HF patients. Uric acid is a by-product of purine metabolism via the xanthine oxidase pathway. Uric acid in its crystallized form can trigger IL-1β-mediated inflammation via activation of the NLRP3 inflammasome. The OPTIME-HF trial evaluated the xanthine oxidase inhibitor oxypurinol, which decreases production of uric acid, in HFrEF patients (LVEF ≤ 40%), but no improvement of morbidity, mortality, or QoL was seen [68].

#### IL-1 Antibodies

In the CANTOS trial, patients with prior MI and hs-CRP levels of > 2 mg/mL were treated with the IL-1β neutralizing antibody canakinumab once every 3 months and had a median follow-up of 3.7 years, leading to less HHF [69]. In a sub-analysis, patients who achieved reduced hs-CRP levels (< 2 mg/L) after 3 months had reduced cardiovascular mortality of 31% [70]. Interestingly, a post hoc analysis of the CANTOS trial showed that IL-1 inhibition reduced HHF- and HF-related mortality linking immunomodulation in HF with improved outcome [69].

Recently, accumulation of unfolded proteins was discovered in both humans and animal models with HFpEF [71], and unfolded protein response (UPR) effectors were found to be decreased in the myocardium of both experimental and human HFpEF [72]. The UPR can activate NF-κB [73] and lead to formation of inflammasomes [74], the latter posing as a new target for HFpEF therapy highlighting the promising nature of canakinumab.

### Methotrexate

Methotrexate is a disease-modifying antirheumatic drug and is primarily used in rheumatoid arthritis. However, it was associated with reduced cardiovascular events, including HHF [75]. In a small prospective randomized clinical trial with HFrEF patients (LV < 45%) treated for 12 weeks, it resulted in a significant reduction of TNF, IL-6, and MCP-1 levels; upregulation of anti-inflammatory cytokines (IL-10 and soluble IL-1 receptor antagonist); and improvement in HF classification, 6-min walk test distance, and QoL. However, treatment did not affect LV remodeling [59, 76]. Methotrexate might be beneficial in the treatment of HFpEF, particularly in patients with a high inflammatory risk profile.

### High-Density Lipoprotein (HDL)/Apolipoprotein A-I (ApoA-I)

High-density lipoprotein (HDL) and its main protein component apolipoprotein A-I (ApoA-I) have immunomodulatory properties, which pose an interesting target in patients with HFpEF [77]. Experimentally, increased ApoA-I/HDL levels showed a protective effect on vascular function by downregulation of the angiotensin-1 receptor [78]. More recently, infusion of recombinant HDL in a TAC model protected against oxidative stress and apoptosis, improving diastolic function compared with sham animals [79], and decreased hypertrophy, fibrosis, and adverse remodeling in murine model of T2DM-induced diabetic cardiomyopathy and HFpEF [80, 81]. The presence of ApoA-I immune complexes, illustrating the presence of autoantibodies, were associated with an increased risk of cardiovascular events [82]. In the MILANO-PILOT trial, recombinant HDL showed no relevant adverse events although the effect on plaque progression was neutral and further drug development was halted. Furthermore, in a large cohort of HF patients, HDL particle analysis showed an association between derangement in small HDL particle concentration and adverse outcome in HFrEF (most pronounced) and HFpEF [83]. To our knowledge, a randomized trial investigating ApoA-I administration or a way to enhance HDL directly has not been performed. In this perspective, proprotein convertase subtilisin/kexin type 9 (PCSK9) inhibitors might come into play, which are safe and seem to increase long-term ApoA-I levels [84].

### B Cell Depletion

B cell responses are crucial in host defense and tightly regulated by our immune system. However, in HFpEF, systemic inflammation combined with exposure of cardiac proteins by damaged or dead cardiomyocytes may break immune tolerance and result in the activation of autoreactive T and B cells [85]. B cell depletion, using monoclonal antibodies such as rituximab (anti-CD20), is used in patients with autoimmune disease and may be interesting in HFpEF. CD20+ B cell depletion significantly improved cardiac function in mice with TAC-induced pressure overload HF, showing reduced dilatation, hypertrophy, fibrosis, and oxidative stress [86]. The anti-inflammatory drug pirfenidone reduced B cell dependent adverse cardiac remodeling [87]. Rituximab was used in six patients with inflammatory dilated cardiomyopathy resistant to steroid treatment and positive for B cells in endomyocardial biopsies. Two injections of rituximab at a 4-week interval on top of standard HF medication resulted in improved LVDD, LVEF, and NYHA class, in five out of six patients [88]. Although the underlying mechanism needs to be elucidated, this shows the potential of B cell targeting therapies.

### Intravenous Immunoglobulin Injection

A key feature of B cells is the production of immunoglobulins, and autoreactive B cells can produce antibodies targeting myocardial proteins [89]. Binding of autoantibodies to cardiac-specific antigens on cardiomyocytes may induce cardiac dysfunction, e.g., by affecting contractility, inducing cellular toxicity and cardiomyocyte lysis [90–93]. The presence of these autoantibodies is associated with end-stage HF [92]. The most abundant immunoglobulin subtype in patients with HF is IgG, including IgG1 and IgG3 subclasses [94]. Intravenous immunoglobulin (IVIG) injection can clear these antibodies from the body, which in HF patient showed to have limited benefit and contradictory results [95, 96]. Monthly IVIG treatment for 6 months was shown to improve LVEF in patients with chronic HF [95], while IVIG treatment did not result in improvement in early onset cardiomyopathy [97]. This suggests that autoantibodies may be more relevant in late stage chronic HF. However, most HF patients showed a decline in LVEF after discontinuation of the therapy [98]. This indicates that treatment should be prolonged or maybe even chronic, with increased risk for adverse side effects.

### Cell Therapy

Progenitor cells, including mesenchymal stromal cells (MSC) and cardiac progenitor cells, are able to improve cardiac function, despite poor cardiac engraftment [99, 100]. Benefits may be directly related to immunosuppressive properties of progenitor cells [101, 102]. MSC and MSC-derived extracellular vesicles have been shown to modulate T and B cells, inhibit the formation of plasma cells, lower antibody production in vitro [101, 103–106], and suppress immunoglobulin production in B cells from HF patients [107]. Moreover, MSC are used as potential immunosuppression for multiple autoimmune disorders and graft-versus-host diseases [108, 109] and could be important to suppress antibody-mediated immune responses in chronic HF. The ongoing REGRESS-HFpEF trial investigates intracoronary treatment with allogenic cardiosphere-derived cells in HFpEF patients and is expected to show reduction in pro-inflammatory and pro-fibrotic signaling (NCT02941705). Experimentally, in a murine T2DM model, MSC injection leads to beneficial immunomodulation and improvement of T2DM-associated diastolic dysfunction and cardiomyocyte stiffness [110].

#### Regulatory T Cells

T cells are found in cardiac tissue of HF patients [93, 111] and have been implicated as important players in the pathogenesis of non-ischemic HF [112]. The amount of regulatory T cells is decreased in patients with HFrEF and HFpEF [113]. In addition, T cell receptor-deficient mice and T cell depletion in wild-type TAC mice reduced adverse remodeling [112]. Regulatory T cells represent a unique subtype of T cells instrumental for the maintenance of immune homeostasis by suppressing T cell responses and cytokine production. Expansion of regulatory T cells, by infusion of IL-2 or IL-2 immune complexes, attenuated LV inflammation and progression of HF in TAC mice [114]. Noteworthy, several clinical trials (phases I–III) using ex vivo expanded regulatory T cells are currently running for different disease entities, mostly solid organ transplantation and autoimmune disease (reviewed in [115]). Although still very preliminary, one may speculate that expansion of regulatory T cells may also be beneficial in HFpEF patients and possibly result in (partially) attenuated disease progression.

## Discussion and Conclusion

Prevalence of HFpEF differs between sexes and races [116], and most patients present with comorbidities linked to chronic inflammation, such as hypertension and T2DM. Based on increasing knowledge of the HFpEF pathophysiology, it is not surprising that previous trials in HFpEF using traditional HF medication did not show benefits on HHF- or HF-related mortality. Therefore, therapies targeting underlying comorbidities and systemic inflammation in early HFpEF might provide better alternatives.

Many questions can be raised on patient selection for HFpEF trials. Given the very heterogeneous HFpEF population, it might not be sensible to aim for a generalized therapy. We should rather consider the sex differences, differences in LVEF, presence of comorbidities, degree of LVDD, and levels of NT-proBNP, troponin, and CRP [11]. As many HFpEF-related comorbidities are more prevalent in women and estrogens play a beneficial role in inflammation and HF progression, postmenopausal women might be more prone to systemic inflammation and HFpEF as reviewed by our group [117]. Interestingly, most trials focusing on immunomodulation in HFpEF still include more men than women (Supplementary Table 2).

The translational challenge to bring promising therapies from experimental models to HFpEF patients as seen with methotrexate use highlights the need for suitable preclinical models, which implements several aspects of the syndrome. Until now, development of new therapies based on underlying disease mechanisms is progressing slowly, as animal models of HFpEF are not yet well established and mimic either one or a selection of risk factors, such as LVH, hypertension, ageing, obesity, or diabetes [118, 119]. Besides, rodent models of LVDD mainly use males and progress to HFrEF within a variable amount of time. This progression from HFpEF to HFrEF does not necessarily occur in humans [120].

Immunomodulation in HFpEF holds encouraging results, but new (immunomodulatory) therapies based on disease mechanisms call for better experimental models for HFpEF. With this review, we aimed to provide an overview of the current state and future perspectives of immunomodulatory therapies for HFpEF based on preclinical experimental models and early clinical data. Promising future therapies include IL-1β antagonists (e.g., anakinra), which show improvement of exercise capacity and QoL and reduce inflammation upon HFpEF [66, 67]. Additionally, canakinumab treatment of patients with prior MI and elevated CRP reduced HHF, and lower CRP was correlated to reduced mortality [69]. However, low-dose methotrexate did not reduce IL-1β, IL-6, or CRP levels [70], which suggests that the ability of the drug to reduce CRP is limited to clinical situations in which inflammation levels are high [70]. As such, future clinical trials may benefit from selecting HFpEF patients with high inflammatory risk.

## Electronic Supplementary Material

ESM 1 (DOCX 24 kb)

## Abbreviations

ApoA-I: Apolipoprotein A-I
CRP: C-reactive protein
cGMP: Cyclic guanosine monophosphate
ECM: Extra cellular matrix
HF: Heart failure
HHF: HF hospitalization
HFpEF: Heart failure with preserved ejection fraction
HFrEF: Heart failure with reduced ejection fraction
HDL: High-density lipoproteins
hs-CRP: High-sensitive C-reactive protein
Ig: Immunoglobulin
IVIG: Intravenous immunoglobulin
IL: Interleukin
ICE: Interleukin converting enzyme
LVDD: Left ventricular diastolic dysfunction
LVEF: Left ventricular ejection fraction
MHCII: Major histocompatibility complex II
NO: Nitric oxide
NLRP3: NOD-like receptor protein
NT-proBNP: N-terminal prohormone of brain natriuretic peptide
PPAR-γ: Peroxisome proliferator-activated receptors
PDE: Phosphodiesterase
PCSK9: Proprotein convertase subtilisin/kexin type 9
PKG: Protein kinase G
QoL: Quality of life
sGC: Soluble guanylate cyclase
TAC: Transverse aortic constriction
TNF-α: Tumor necrosis factor
T2DM: Type 2 diabetes mellitus
MSC: Mesenchymal stromal cells
