# Supplementary material for: Immunomodulation in Heart Failure with Preserved Ejection Fraction: Current State and Future Perspectives
Source: J Cardiovasc Transl Res. 2020 May 22;14(1):63–74. doi: 10.1007/s12265-020-10026-3 (PMC7892675; doi:10.1007/s12265-020-10026-3)
Supplement: Supplementary file 1 — (DOCX 24 kb) [file 12265_2020_10026_MOESM1_ESM.docx]

**Supplementary Material for Kessler et al: Immunomodulation in heart failure with preserved ejection fraction: current state and future perspectives**

| **Supplementary Table 1. Overview of Phase III randomized clinical trials in HFpEF patients** | | | | | | |
| --- | --- | --- | --- | --- | --- | --- |
| **Drug (Trial)** | **Year** | **Study size,**  **(% men)**  **LVEF** | **Patient characteristics** | **Results** | **Effect on inflammation** | **Reference DOI** |
| Sacubitril/Valsartan 97/103mg  (PARAGON-HF) | 2019 | N=4822  (48.2%)  LVEF≥45% | NYHA II-IV, HHF <9months, mean age 72.8 years  Median NT-proBNP 915 pg/ml  Hypertension 95.6%  T2DM 43.5%  Exclusion: eGFR <30ml/min/1.73m^2^ | No difference in mortality or HHF | Not reported | Solomon S.D. et al. 2019  10.1056/NEJMoa1908655 |
| Spironolacton  45mg  (TOPCAT) | 2014 | N=3445  (48.6%)  LVEF≥45% | NYHA II-IV, HF diagnosis <12 months  Mean age 68.7 years  Median NT-proBNP 1017 pg/ml  Hypertension not reported  T2DM not reported  Exclusion: eGFR <30ml/min/1.73m^2^ | No difference in mortality or HHF | No difference in hs-CRP | Pitt B. et al. 2014  10.1056/NEJMoa1313731  Myhre, P. L. et al. 2020  10.1161/CIRCHEARTFAILURE.119.006638 |
| Carvedilol  10mg  (J-DHF) | 2014 | N=245  (49%) LVEF>40% | NYHA II-IV, HHF <12 months  Mean age 73 years  Median BNP 219 pg/ml  Hypertension 80%  T2DM 30%  Exclusion: eGFR <30ml/min/1.73m^2^ | No difference in mortality or HHF | Not reported | Yamamoto K. et al. 2014  10.1093/eurjhf/hfs141 |
| Irbesartan  300mg (I-PRESERVE) | 2008 | N=4128  (41%)  LVEF≥45% | NYHA II-IV, HHF <6 months  Mean age 72.7 years  Median NT-proBNP 360 pg/ml  Hypertension 88%  T2DM 28%  Exclusion: eGFR <30ml/min/1.73m^2^ | No difference in mortality or HHF | Not reported | Massie et al. 2008  10.1056/NEJMoa0805450 |
| Perindopril  4mg (PEP-CHF) | 2006 | N=850  (46%)  LVEF>40% | NYHA II-IV, HHF <6 months  Mean age 75 years  Median NT-proBNP 355 pg/ml  Hypertension 79%  T2DM 21%  Exclusion: eGFR <30ml/min/1.73m^2^ | No difference in mortality or HHF | Not reported | Cleland et. Al. 2006  doi.org/10.1093/eurheartj/ehl250 |
| Digoxin  Varying dose (DIG) | 1997 | N=988  (52.6%)  LVEF≥45% | NYHA II-IV, HF criteria  Mean age 67.3 years  Median NT-proBNP not specified  Hypertension 62.5%  T2DM 29.1%  Exclusion: eGFR <30ml/min/1.73m^2^ | No difference in mortality or HHF | Not reported | Digitalis Investigation Group 1997  10.1056/NEJM199702203360801  Abdul-Rahim A.H. et al. 2018  10.1002/ejhf.1160 |
| Nebivolol  10mg  (SENIORS) | 2005 | N=2128  (61.6%) LVEF>35% (subgroup analysis) | NYHA II-IV, HHF <12 months  Mean age 76.1 years  Median NT-proBNP not specified  Hypertension 62.3%  T2DM 26.9%  Exclusion: eGFR <30ml/min/1.73m^2^ | No difference in mortality or HHF | Not reported | Flather M.D. 2005  10.1093/eurheartj/ehi115 |
| Candesartan  32mg  (CHARM-Preserved) | 2003 | N=3023  (61%) LVEF>40% | NYHA II-IV, HHF in the past  Mean age 67 years  Median NT-proBNP not reported  Hypertension 65%  T2DM 28%  Exclusion: LVEF≤40% | No difference in mortality or HHF | Not reported | Yusuf S. et al. 2003 doi.org/10.1016/S0140-6736(03)14285-7 |

Abbreviations: HFpEF = heart failure with preserved ejection fraction; HF = heart failure; HHF = heart failure hospitalization; LVEF = left ventricular ejection fraction; ARB = angiotensin receptor blockers; NT-proBNP = N-terminal prohormone of brain natriuretic peptide; LVDD = left ventricular diastolic dysfunction; SGLT2 inhibitor = sodium glucose co-transporter 2 inhibitor; hs-CRP = high-sensitive C-reactive protein; eGFR = estimated glomerular filtration rate.

| **Supplementary Table 2. Ongoing clinical trials for immunomodulation in HF(pEF)** | | | |
| --- | --- | --- | --- |
| **Trial** | **Drug** | **Patient population** | **Reference**  **DOI** |
| RELAX | sildenafil | N=216 (52% men) stable outpatients with HFpEF.  Double-blind, placebo-controlled randomized clinical trial. | Redfield M. et al. 2013  10.1001/jama.2013.2024 |
| NEAT-HFpEF | isosorbide | N=110 (43% men) with HFpEF and age >50 years.  Multi-center, double-blind, cross-over study. | Redfield M. et al. 2015  10.1056/NEJMoa1510774 |
| INDIE-HFpEF | inorganic nitrite | N=105 (44% men) with HFpEF.  Multicenter, double-blind, placebo-controlled, 2-treatment, crossover trial. | Bolraug B. et al. 2018  10.1001/jama.2018.14852 |
| DILATE-1 | riociguat | N=21 (39% men) with HFpEF and pulmonary hypertension (clinically stable patients receiving standard HF therapy with LVEF>50%, mean pulmonary artery pressure ≥ 25 mm Hg, and pulmonary arterial wedge pressure > 15 mm Hg).  Multicenter, double-blind, randomized, placebo-controlled, parallel-group phase IIa study. | Bonderman D. et al. 2014  10.1378/chest.14-0106 |
| SOCRATES-PRESERVED | vericiugat | N=477 (52% men) with HFpEF.  Prospective, randomized, placebo-controlled double-blind, Phase IIb dose-finding study. | Pieske B. et al. 2017  10.1093/eurheartj/ehw593 |
|  | pentoxifylline | N=18 (62% men) with decompensated congestive HF secondary to idiopathic dilated cardiomyopathy.  Prospective, randomized, double-blind, placebo-controlled study. | Silwa K. et al. 2002  10.1016/s0002-9149(02)02779-0 |
|  | thalidomide | N=7 (100% men) with advanced HFrEF, LVEF<40% and circulating TNF>3.0pg/mL. | Agoston I. et al. 2002  10.1054/jcaf.2002.128684 |
|  | thalidomide | N=56 (75% men) with HFrEF and LVEF<40%.  Double-blind, placebo-controlled study. | Gullestad L. et al. 2005  10.1161/CIRCULATIONAHA.105.564971 |
| RECOVER/ RENAISSANCE | etanercept | N=1500 with HFpEF.  Discontinued. | Mann D.L. et al. 2015  10.1161/CIRCRESAHA.116.302317 |
| ATTACH | infliximab | N=150 (82% men) with stable NYHA class III or IV HF and LVEF <or=35%.  Randomized, Double-blind, placebo-controlled, pilot trial. | Chung E. S. et al. 2003  10.1161/01.CIR.0000077913.60364 |
| D-HART | anakinra | N=12 (8% men) with HFpEF and plasma CPR>2 mg/L.  double-blind, randomized, placebo-controlled, crossover trial. | Van Tassel B.W. et al. 2014  10.1016/j.amjcard.2013.08.047 |
| D-HART2 | anakinra | N=31 (36% men) with with HFpEF and plasma CPR>2 mg/L.  double-blind, randomized, placebo-controlled, crossover trial. | Van Tassel B.W. et al. 2018  10.1161/CIRCHEARTFAILURE.118.005036 |
| CANTOS | canakinumab | N=10 061 (75% men) with prior myocardial infarction and hs-CRP>2 mg/mL.  Randomized, placebo-controlled trial. | Everett B.M. et al. 2019  10.1161/CIRCULATIONAHA.118.038010 |
| OPTIME-HF | oxypurinol | N=405 (73% men) with HFrEF and LVEF<40%.  Multicenter, randomized, double-blind, placebo-controlled, parallel group study. | Hare J.M. 2008  doi:10.1016/j.jacc.2008.01.068 |
| METIS | methotrexate | N=50 (79% men) with ischemic HFrEF and LVEF<45%.  Prospective randomized, double-blind clinical trial. | Moreira D.M. et al. 2009  10.1016/j.cardfail.2009.06.439 |
|  | rituximab | N=6 (67% men) with inflammatory dilated cardiomyopathy resistant to steroid treatment and positive for B-cells in endomyocardial biopsies.  Case report. | Tschöpe C. et al. 2019  10.1093/ehjcr/ytz131 |
|  | IVIG | N=40 (83% men) with NYHA functional class II to IV congestive HF and LVEF<40%.  Randomized and double-blind study. | Gullestad L. et al. 2001  10.1161/01.cir.103.2.220 |
| IMAC | IVIG | N=62 (23% men) with early onset cardiomyopathy and and LVEF<40%.  Prospective placebo-controlled trial. | Mc Namara D.M. et al. 2001  10.1161/01.cir.103.18.2254 |
| REGRESS-HFpEF | cardiosphere-derived cells | N=40 (estimated) with HFpEF.  Randomized, double blind, placebo-controlled study. | NCT02941705 (ongoing) |

Abbreviations: HFpEF = heart failure with preserved ejection fraction; HF = heart failure; LVEF = left ventricular ejection fraction; NYHA = New York Heart Association; TNF= Tumor Necrosis factor; hsCRP = high sensitive C reactive protein; IVIF = intravenous immunoglobulin; N indicates numerb of patinets included in the study/trial; DOI = Digital Object Identifier.
